# SV40 intron, a potent strong intron element that effectively increases transgene expression in transfected Chinese hamster ovary cells

**DOI:** 10.1111/jcmm.13504

**Published:** 2018-02-14

**Authors:** Dan‐hua Xu, Xiao‐yin Wang, Yan‐long Jia, Tian‐yun Wang, Zheng‐wei Tian, Xin Feng, Yin‐na Zhang

**Affiliations:** ^1^ Department of Biochemistry and Molecular Biology Xinxiang Medical University Xinxiang Henan China; ^2^ Pharmacy collage Xinxiang Medical University Xinxiang Henan China; ^3^ Grade 2014 The Third Clinical Medical College of Xinxiang Medical University Xinxiang Henan China

**Keywords:** gene expression, intron, Chinese hamster ovary (CHO), gene regulation

## Abstract

Chinese hamster ovary (CHO) cells have become the most widely utilized mammalian cell line for the production of recombinant proteins. However, the product yield and transgene instability need to be further increased and solved. In this study, we investigated the effect of five different introns on transgene expression in CHO cells. hCMV intron A, adenovirus tripartite leader sequence intron, SV40 intron, Chinese hamster EF‐1alpha gene intron 1 and intervening sequence intron were cloned downstream of the eGFP expression cassette in a eukaryotic vector, which was then transfected into CHO cells. qRT‐PCR and flow cytometry were used to explore eGFP expression levels. And gene copy number was also detected by qPCR, respectively. Furthermore, the erythropoietin (EPO) protein was used to test the selected more strong intron. The results showed that SV40 intron exhibited the highest transgene expression level among the five compared intron elements under transient and stable transfections. In addition, the SV40 intron element can increase the ratio of positive colonies and decrease the coefficient of variation in transgene expression level. Moreover, the transgene expression level was not related to the gene copy number in stable transfected CHO cells. Also, the SV40 intron induced higher level of EPO expression than IVS intron in transfected CHO cell. In conclusion, SV40 intron is a potent strong intron element that increases transgene expression, which can readily be used to more efficient transgenic protein production in CHO cells.

## Introduction

The production of certain types of recombinant proteins requires the use of animal cells such as Chinese hamster ovary (CHO) cell lines because CHO cells confer some advantages, such as post‐translational modifications and glycosylation of glycoproteins produced by CHO cells are more human‐like, with the absence of immunogenic α‐galactose epitope and capable of adapting and growing in suspension culture 1, 2, 3. In the past 20 years, a great efforts have been made to increase the productivity of recombinant protein, and the productivity of recombinant protein has reached 10–15 g/l in Mab and Fc‐fusion protein production 4, which was mainly resulted from improvements of cell line development through vector optimizing, effective selection methods and media optimization 5, 6. Expression vector plays a key role in recombinant protein production, and considerable efforts were made to optimize CHO expression vector. Several *cis*‐acting elements, such as matrix attachment regions (MAR), ubiquitous chromatin opening elements (UCOE), stabilizing and antirepressor elements (STAR), insulators and introns, have been utilized in vector engineering to achieve stable cell lines with high expression 7, 8, 9, 10, 11.

Introns, as a major part of non‐coding sequences, can regulate gene expression in a variety of ways except increasing the coding capacity of the genome *via* alternative splicing 12. It has been demonstrated that several introns can increase transgene expression in different mammalian cell lines, such as CHEFI, CMVI and hBG introns 13, 14, 15, but there was the contradictory report regarding the intron's effect on transgene expression 16.

Introns confer the potential to improve gene expression in eukaryotes; however, there are little reports regarding introns positioned downstream of the expression cassette on transgene expression, especially in CHO cell, and the systematic research of effect of introns on transgene in CHO cells was not reported. In this study, we systematically evaluated the effects of five introns on transgene expression in transfected CHO cells and try to develop a highly efficient mammalian expression vector for recombinant protein production.

## Materials and methods

### Introns and constructs

**Figure 1:**
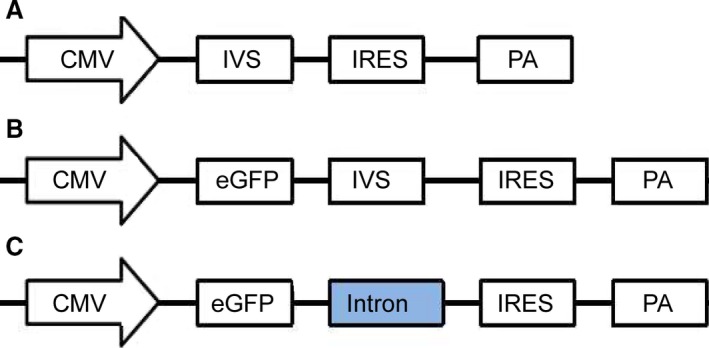
Representation of the constructs used in this study. (**A**) Mammalian cell expression vector—pIRES‐neo (Gene bank:U89673) containing CMV promoter, synthetic intron (IVS) and internal ribosome entry site(IRES). (**B**) eGFP sequence was obtained from the pEGFP‐C1 vector and cloned downstream CMV promoter of pIRES‐neo vector to produce the pIRES‐EGFP vector. (**C**) Four different intron elements, including hCMVI, TPLI, SVI and CHEF1 gene intron1, were artificially synthesized by Sangon Biotech Co., Ltd. and replaced the IVSI of pIRES‐EGFP vector by fusion cloning technique to produce the vectors containing different introns. CMV, human cytomegalovirus promoter; *eGFP*, enhanced green fluorescence protein; IVS: synthetic intron; IRES, internal ribosome entry site; and poly A, polyadenylic acid. Intron: include hCMV intron A (hCMVI), TPL intron(TPLI), SV40 intron(SVI) and CHEF1 gene intron1.

pIRES‐neo(Gene bank:U89673) containing the CMV promoter and synthetic intron (IVS) was used as the original vector. The enhanced green fluorescent protein (eGFP)‐coding sequence was obtained from the pEGFP‐C1 vector (Gene bank: U55763) and cloned into pIRES‐neo vector to generate pIRES‐EGFP vector. Four different intron elements, including hCMV intron A (hCMVI), TPL intron (TPLI), SV40 intron (SVI) and CHEF1 gene intron1 (CHEFI), were artificially synthesized by Sangon Biotech Co., Ltd. (Shanghai, China) and replaced the IVSI of pIRES‐EGFP vector (*i.e*. the IVS was excised and replaced with different intron elements by fusion cloning technique to produce the vectors containing different introns; Fig. 1). The intron element sequences are listed in Fig. S1.

### Cell culture and transfection

CHO‐S cells (Life Technologies # A11557‐01, Carlsbad, CA, USA) were grown at 37°C in a humidified incubator with 5% CO_2_, in Dulbecco's modified Eagle's medium (Gibco, Grand Island, NY, USA) supplemented with 10% foetal bovine serum (Gibco) for adherent culture. One day before transfection, cells were seeded into 24‐well plates at approximately 2 × 10^5^ cells/well. After the cells reached 80–90% confluence, cells were transfected with the above five vectors using Lipofectamine 3000 reagent (Invitrogen, Carlsbad, CA, USA) according to the instructions of manufacturer.

### Generation of stable transfected cell lines

After 48 hrs transfection, stable transfected cells were selected by 800 μg/ml geneticin (G418, Invitrogen) for 2 weeks until positive colonies appeared. Then, stable cell populations exhibiting stable transgene integration were cultured in CD CHO medium (Life Technologies # 10743‐029) supplemented with 8 mM l‐glutamine (Life Technologies # 25030‐024) in 125‐ml Corning shake flasks (Sigma‐Aldrich #431255, Wisconsin, USA) with 30 ml medium with the presence of 500 μg/ml G418 for 30 days and then collected for further analysis. For all constructs, triplicate experiments were performed for each independent experiment.

### Flow cytometry

Chinese hamster ovary cells were obtained by screening at different time‐points after the transfection. After 48 hrs transfection, the transfection efficiency and eGFP mean fluorescence intensity (MFI) of each vector were analysed using a FACSCalibur cytometer (Becton Dickinson, Franklin Lakes, NJ, USA), the untransfected cells used as the negative control. A total of 100,000 fluorescent events were acquired using a 530/15 bandpass filter for the eGFP signal, which was obtained with fluorescence emission centred at 530 nm. After 30 day transfection, the MFI for each vector was measured again, and lower, medium, higher producers (% M1, M2, M3) and coefficient of variation (CV) of each sample were determined at the same time using a FACSCalibur cytometer. All the experiments were repeated three times.

### Real‐time quantitative PCR

Genomic DNA and total RNA were isolated from 5 × 10^6^ cells using the Genomic DNA Mini Preparation Kit (Beyotime, Shanghai, China) and RNApure Tissue Kit (Beijing ComWin Biotech Co., Ltd., Beijing, China), respectively. Analysis of the relative eGFP gene copy numbers and mRNA levels was determined using real‐time quantitative PCR (qRT‐PCR) at day 30 after transfection. Primers were designed according to the eGFP sequence, forward primer: 5′‐GCTGGTTTAGTGAACCGTCAG‐3′ and reverse primer: 5′‐AGGTGGCATCGCCCTCGCCC‐3′. Glyceraldehyde phosphate dehydrogenase (GAPDH) gene was used as an internal reference; the primers were designed as follows: 5′‐CGACCCCTTCATTGACCTC‐3′(forward primer) and 5′‐CTCCACGACATACTCAGCACC‐3′ (reverse primer). According to the manufacturer′s instructions (Takara Bio, Dalian, China), the PCR was carried out. The cycling parameters were as follows: 95°C for 3 min.; 30 cycles of 94°C for 30 sec., 50°C for 30 sec. and 72°C for 30 sec.; and 60°C for 5 min. The 2^−ΔΔCt^ method was used for calculating relative eGFP copy numbers and the level of eGFP mRNA.

### Expression of EPO

To further investigate the role of the selected strong intron on recombinant protein production, the expression vector containing erythropoietin (EPO) was constructed according to the previous report 17. ELISA and Western blot analyses were performed to analyse EPO expression level. Twenty stable clones transfected with EPO‐containing vector were picked out, and cells were suspended. Volumetric EPO production (mg/l) for initial CHO‐EPO cell lines cloned was scaled up to 125 ml Corning shake flasks. Cells were grown in serum‐free, protein‐free, CD CHO medium supplemented with 8 mM l‐glutamine. When the cell number reached 8 × 10^6^/ml, cells supernatant was collected for EPO analysis by ELISA and Western blot according to previously described 17.

### Statistical analysis

All experiments were carried out triplicates, and data are reported as mean ± standard deviation (S.D.). After analysis of variance, multiple comparison procedure was further performed to assess pairwise differences in expression confirmed by analysis of variance. Results with *P* values of less than 0.05 were considered statistically significant. All statistical analyses were carried out with the SPSS 18.0 software (SPSS Inc., Chicago, IL, USA).

## Results

### Transfection efficiency and transient expression

**Figure 2:**
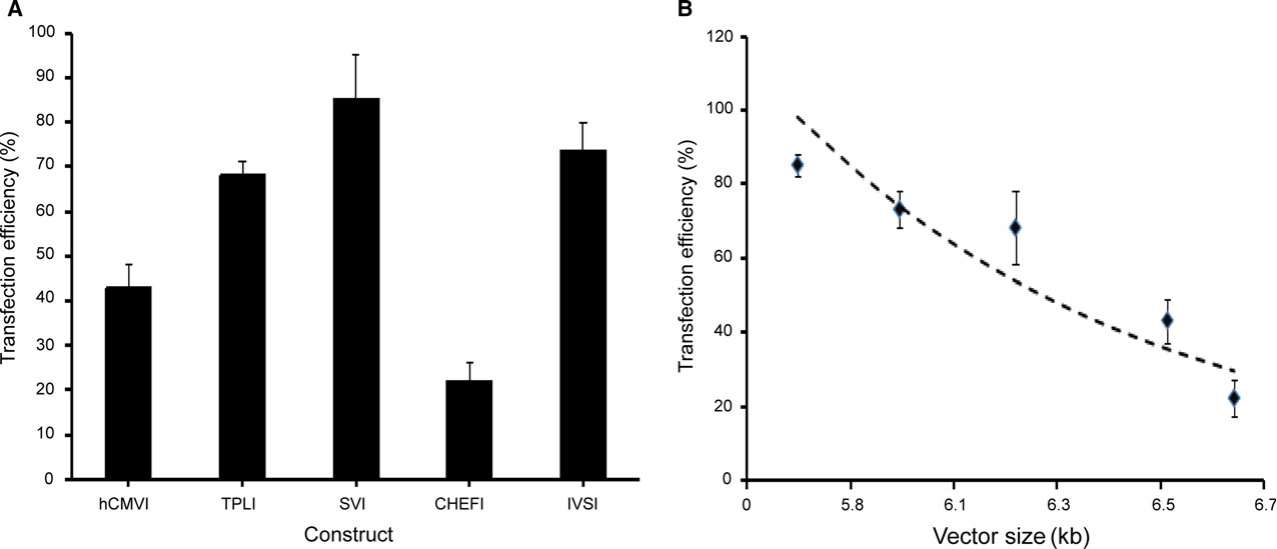
Analysis of transfection efficiency and correlation between transfection efficiency and vector size. The five constructed vectors were transfected into CHO cells using Lipofectamine^®^ 3000 Transfection Reagent. The transfection efficiency was analysed using a FACSCalibur cytometer. (**A**) Analysis of the transfection efficiency. Three independent experiments were performed in this study. Standard error of the mean (S.E.M.) is indicated. (**B**) Correlation between transfection efficiency and vector size. Transfection efficiency analysed by FACSCalibur cytometer decreased exponentially as vector size increased. The graph was created with Microsoft Office Excel.

In this work, the effect of different introns on transfection efficiency was first evaluated using eGFP as a reporter gene. Our results showed that transfection efficiency in CHO cells was highest for SVI (85.3%), followed by the IVSI, TPLI, hCMVI and CHEFI (73.8%, 68.1%, 43.2%, 22.1%; Fig. 2A).

Given each vector contained different *cis*‐acting elements, their sizes were different. Therefore, we evaluated the correlation between vector size and transfection efficiency. The results showed that the transfection efficiency was generally reduced as vector size increased (Fig. 2B). SVI showed the highest transfection efficiency with the minimum length, whereas the longest intron (CHEFI) exhibited the lowest transfection efficiency.

**Figure 3:**
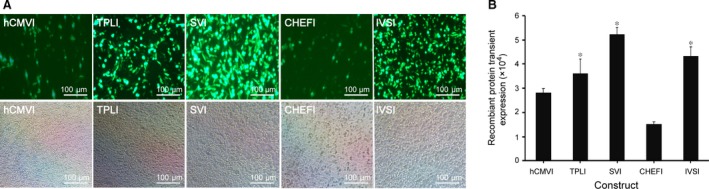
Recombinant protein transient expression of different intron elements. The constructed vectors were transfected into CHO cells, and eGFP expression levels were estimated by fluorescence microscopy at 48 hrs after transfection and recombinant protein transient expression was determined at 48 hrs after transfection with the FACSCalibur. (**A**) eGFP transient expression observed by fluorescence microscopy. (**B**) Analysis of the transient expression of different intron elements. Three independent experiments were performed in this study. These results are the mean values obtained for three independent experiments. Standard error of the mean (S.E.M.) is indicated (Student's *t*‐test, *P <* *0.05).

Next, the effect of different introns on transient gene expression was evaluated. eGFP transient expression was highest in cells transfected with vectors containing SVI, followed by IVSI, TPI, hCMVI and CHEFI. The eGFP transient expression of cells transfected with the vectors containing the SVI, IVSI and TPLI was significantly higher than that of hCMVI and CHEFI (*P *<* *0.05; Fig. 3A and B).

### Ratio of positive colonies in stable transfected cells

**Figure 4:**
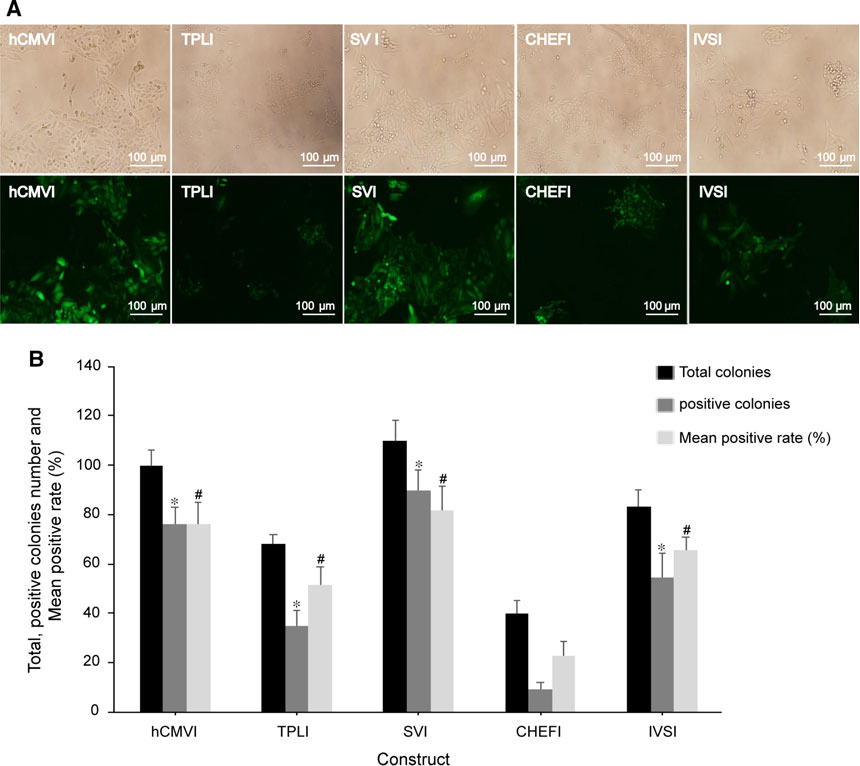
The ratio of positive colonies of different intron elements. At 48 hrs after transfection, G418 was added to CHO cells and cultured for 10–14 days until a single colony appeared. The cell colonies number was observed in bright field, and the total positive colonies number and mean positive ratio were calculated based on white light (upper lane) and fluorescence (under lane). (**A**) Scale bars: 100 μm; (**B**) statistical analysis result of the total positive colonies number and mean positive ratio (%). These results are the mean values obtained for three independent experiments, standard error of the mean (S.E.M.) is indicated (Student's *t*‐test, *P <* *0.05; ^#^ *P *<* *0.05).

Selection of high‐level expression of positive colonies ratio in CHO cells typically requires the laborious and time‐consuming procedure. During this selection process, the differences between different vectors were reflected in the ratio of positive colonies and growth characteristics. In this study, we evaluated the introns on the positive colonies ratio of stable transfected CHO cells. In cells transfected with vectors harbouring SVI, hCMVI, IVSI and TPLI yielded more ratios of positive colonies and higher eGFP expression observed by fluorescence microscopy compared with CHEFI intron element (Fig. 4A). The ratio of high‐level eGFP expression of vector containing SVI, hCMVI, IVSI and TPLI elements achieved the 81.8%, 76.0%, 65.6% and 51.5%, respectively, while that of CHEFI only achieved 22.5% (Fig. 4B). The positive colonies number and ratios of positive colonies transfected with the vectors containing the SVI, IVSI, TPLI and hCMVI were significantly higher than that of CHEFI (*P *<* *0.05, Fig. 4B). In addition, cells transfected with vectors harbouring SVI grew more rapidly than those transfected with the other intron elements.

### Analysis of stable transgene expression

Chinese hamster ovary cells were transfected with the vectors followed by G418 selection to establish stable transfectants. After 30 day transfection, the eGFP mRNA was analysed using qPCR, and MFI of eGFP was measured by flow cytometry.

**Figure 5:**
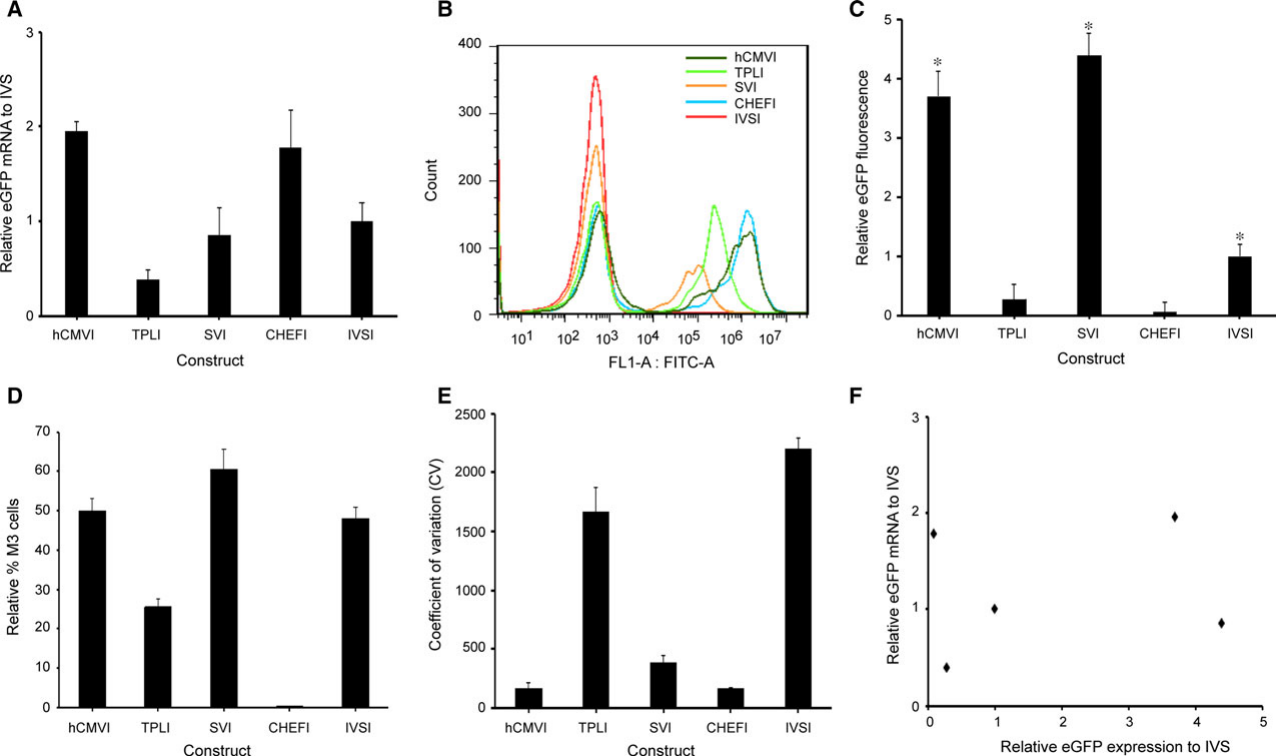
Effect of different introns on the production of eGFP in stable transfected CHO‐S cell. (**A**) Relative eGFP mRNA levels in stable transfected cells. At 48 hrs after transfection, stable transfected cells were selected by 800 μg/ml geneticin selection for 2 weeks until positive colonies appeared. Then, stable cell populations exhibiting stable transgene integration were cultured in CD CHO medium supplemented with 8 mM l‐glutamine in 125‐ml Corning shake flasks with 30 ml medium with the presence of 500 μg/ml G418 for 30 days. Total RNA was isolated from 5 × 10^6^ cells using the RNApure Tissue Kit, analysis of the mRNA levels was determined using real‐time quantitative PCR. (**B**) eGFP MFI was determined by cytometry for stable transfected cell pool with the different introns. Cells were collected and the eGFP fluorescence profile was measured for by FACSCalibur at 30 days after transfection. (**C**) eGFP MFI expression was normalized to IVS, and in the statistical analysis of eGFP expression, fold change values were normalized to those of the IVS, whose value was set to 1. Three independent experiments were performed in this study. Standard error of the mean (S.E.M.) is indicated (Student's *t*‐test, *P <* *0.05). (**D**) Percentage of high producers (% M3), fluorescence values greater than 10^4^. Three independent experiments were performed in this study. Standard error of the mean (S.E.M.) is indicated. (**E**) Coefficient of variability (CV). CV values are expected to reflect variations in transgene expression. The results are the mean values obtained for 3 independent experiments, standard deviation is indicated. (**F**) Correlation between the relative eGFP mRNA levels and relative eGFP protein expression in stable transfected CHO cell. Little correlation was noted between the eGFP mRNA levels and the eGFP protein.

The relative eGFP mRNA level was highest in cells transfected with vectors with hCMVI, followed by CHEFI, IVSI, SVI and TPLI (Fig. 5A). When the eGFP mRNA level from vector containing the IVSI was set as 1.0, the expression levels in CHO transfected with vector containing the SVI, hCMVI, TPLI and CHEFI were 0.85, 1.92, 0.34 and 1.78, respectively.

Among constructs containing the five introns, SVI induced the highest eGFP protein expression, followed by the hCMVI, IVSI, TPLI and CHEFI (Fig. 5B). hCMVI showed lower transient expression but higher stable expression. Stable expression of the eGFP protein of the vectors with the SVI, hCMVI and IVSI was significantly higher than those of vectors with TPLI and CHEF1 gene intron1 (*P *<* *0.05, Fig. 5B). When the eGFP protein expression level from vector containing the IVSI was set as 1.0, the expression levels in CHO transfected with vector containing the SVI, hCMVI, IVSI, TPLI and CHEFI were 4.39, 3.69, 0.18 and 0.008, respectively. The eGFP protein expression of SVI, hCMVI was significantly higher than that of IVSI, while the expression level of TPLI and CHEFI was remarkably reduced, suggesting that the intron elements can regulate the transgene expression significantly (Fig. 5C). Analysis of the total population of eGFP protein expressing cells indicated that the SVI, hCMVI, IVSI and TPLI resulted in significantly higher percentages of highly expressing cells (% M3) when compared with the CHEFI (Fig. 5D). Interestingly, we found that several introns showed the lower of the coefficient of variation (CV) of eGFP protein expression level (Fig. 5E). CV values are expected to reflect variations in transgene expression; CV was lower in the vector with SVI and hCMVI, IVS intron and TPLI than that in CHEF1 gene intron1. We conclude that SVI elements can decrease the variability of transgene expression in stable transfected CHO cells.

Analysis of the relationship between the transgene expression and mRNA levels revealed that the highest expression of SVI was estimated at a relative lower mRNA levels, and the lower expression of CHEFI was estimated at a relative higher mRNA levels (Fig. 5F). The results suggested that the protein expression level of eGFP was not related to the mRNA levels in stable transfected CHO cell pool (Fig. 5F).

### Analysis of transgene copy number

**Figure 6:**
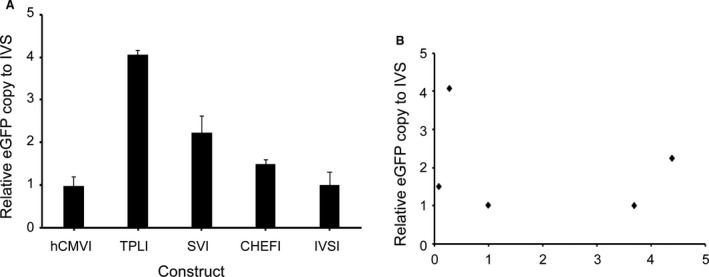
(**A**) Relative eGFP copy number in stable transfected cells. Fluorescent quantitative PCR was used to measure relative eGFP gene copy numbers. The 2^−ΔΔCt^ method was used to calculate relative eGFP copy numbers. The eGFP gene copy numbers were normalized to the IVS whose value was set to 1. Three independent experiments were performed in this study. Standard error of the mean (S.E.M.) is indicated. (**B**) Correlation between the relative eGFP copy and relative eGFP expression in stable transfected CHO cell. Little correlation was noted between the eGFP copy and the eGFP expression.

To investigate the relationship between the transgene expression and transgene copy number, the number of transgene copies per cell for each vector was analysed using qPCR. Each analysed result was normalized to the IVSI, the highest expression of SVI was estimated at a relative lower copy number, and the vector with TPLI was estimated at a higher copy number (2.31 *versus* 4.25, Fig. 6A). The results suggested that the expression level of eGFP was not related to the gene copy number in stable transfected CHO cell pool (Fig. 6B).

### EPO expression

**Figure 7:**
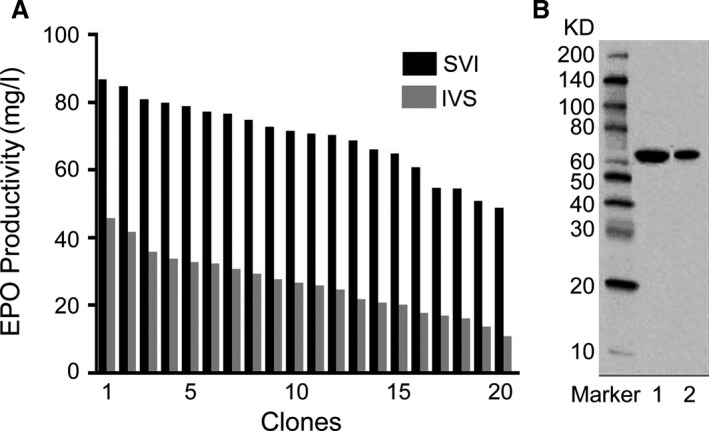
Analysis of EPO protein expression by ELISA and Western blot. The vectors containing EPO were transfected into CHO cells, and 20 single cell clones were picked out. Cells supernatant was collected for analysis. (**A**) Analysis of volumetric EPO production (mg/l) by ELISA. (**B**) Western blot analysis. Lane 1, SV40 intron; 2, IVS intron.

We assessed the effect of SV40 intron on the secreted protein expression by analyse the level of EPO in CHO cells in suspension, serum‐free medium culture. We isolated 20 cell clones by limiting dilution using either the SVI or IVSI and analysed EPO expression by using ELISA and Western blot analyses. ELISA results showed that higher EPO expression occurred at SVI, the mean expression level was 68.9 ± 3.2 mg/l (Fig. 7A), the highest production of EPO in the single cell clone was 86.3 mg/l, and the expression level was 2.72‐fold compared to the IVS. In addition, the protein expression was also confirmed by Western blot results, and Western blot results indicated that EPO expression mediated by the SVI was higher than that of IVS (Fig. 7B).

## Discussion

The expression of recombinant protein in mammalian cells is often low and unstable. It has been known that the level of expression of transgene from a mammalian expression system correlates mainly with the expression vector, so that the use of an optimal expression vector is a primary requirement for high‐level expression of transgene in mammalian cells. Expression vector is usually controlled by regulatory elements 18, 19, 20, which usually exists in non‐coding regions, such as upstream enhancers and promoters and polyadenylation elements and terminator in downstream regions of the genes 21, 22, 23, 24. Introns, as non‐coding DNA sequences, have the function to regulation gene expression in eukaryotes by a variety of mechanisms, such as enhancing RNA polymerase II process activity, promoting the interaction between splicing proteins and certain transcription factors, connecting and facilitating multiple types of RNA processing mechanisms and affecting nuclear mRNA export, translational efficiency and RNA decay 25, 26, 27. Because of CMV has high efficiency in a broad range of cell types and become the most popular promoter for recombinant protein expression 28, its potency can be further enhanced by the presence of its downstream intron 29, 30.

In the present study, we compared the effect of five intron elements positioned downstream regions of the genes on transgene expression levels in transfected CHO cells and found that SVI is a potent strong intron element that increases transgene expression in transfected CHO cells. We first used eGFP reporter gene which is frequently used to evaluate the regulation effect of cis‐acting element. eGFP gene was usually first used to study the effect of cis‐acting element on transgene expression and then the genes of interest (GOI), such as antibody and other proteins were further studied. The previous studies showed that GOI expression level was consistent with eGFP reporter gene 31, 32, 33.

We found that the SVI intron elements can produce the higher transfection efficiency. The transfection efficiency is influenced by the size of the vector to some extent, but the structure and configuration of vector had significant effects on transfection efficiency. In the present study, the size of five intron elements used hCMVI, TPLI, IVSI, SVI and CHEFI was as follows: SVI < IVSI < TPLI < hCMVI < CHEFI. However, the SVI showed the highest transfection efficiency, followed by IVSI, TPLI, hCMVI and CHEFI. The results were consistent with the results of some other cis‐acting elements 34.

In stable transfections, as compared to the IVS intron, vectors containing the SV40 intron enhance eGFP expression levels by approximately 4.39‐fold in the CHO cell, and the effect of other introns was consistent with the results in transient transfections. It has been reported that several intron elements can improve transgene expression in mammalian animal cell system, such as the intron A of hCMV had positive effect on gene expressions in both transient and stable transfections in monkey kidney cells, human foreskin fibroblast, HeLa cells, CHO‐K1 and HEK 293 cells 35, 36, 37. Human EF‐1α first intron could enhance the level of gene expression in HeLa and CHO cell lines 38; application of CHEF1 gene intron1 to achieve higher transgene expression has been demonstrated previously by Running *et al*. 39. In general, these reports demonstrated that intron positioned upstream of the expression cassette exerted high‐level gene expression. Our results first demonstrated that SVI can improve transgenic expression in CHO cells and demonstrated that hCMVI and SVI positioned downstream of the expression cassette can enhance transgene expression. However, in this study, compared to IVS intron, TPL intron and CHEF1 gene intron1 did not result in enhancing transgene; the regulation function of different introns is diverse; and the reason may be related to the type of cell line, vector, promoter and component of introns. For example, TPL introns and CHEFI contain much more transcription factor binding (TFB) motifs but did not result in enhancing transgene expression downstream of the expression cassette, the findings led us to propose that the introns contain larger number of TFB motifs may be suitable for positioning upstream of the expression cassette. SV40 intron contains only 99nt and has been found to increase splicing efficiency and transport much more efficient mRNA 40, 41, 42. In this study, SV40 intron positioned downstream of the expression cassette results in highest transgene expression, this result may be relative to the function of cryptic splicing, increasing the poly A tail‐length, increasing the half‐life of mRNA and translation rate.

To further investigate the effect of strong intron on the expression of gene of interest, we evaluated the expression of therapeutic secreted protein EPO, mediated by the SV40 and IVS intron under serum‐free medium culture conditions. Consistent with eGFP gene expression, EPO expression level in CHO cells transfected with the vector containing SV40 intron was significantly higher than of that of cells transfected with the vector containing the IVS intron.

In conclusion, we first systematically investigated the effect of five introns on transgene expression in transfected CHO cells and find that SV40 intron can effectively increase transgene expression, which may be a very useful regulatory element for high‐level expression of recombinant proteins in CHO cells.

## Conflict of interest

All authors have no conflict of interest regarding this manuscript.

## Supporting information


**Fig. S1** Cis‐acting elements sequences used in this study
