# Supplementary material for: SV40 intron, a potent strong intron element that effectively increases transgene expression in transfected Chinese hamster ovary cells
Source: J Cell Mol Med. 2018 Feb 14;22(4):2231–9. doi: 10.1111/jcmm.13504 (PMC5867124; doi:10.1111/jcmm.13504)
Supplement: Supplementary file 1 — Fig. S1 Cis‐acting elements sequences used in this study [file JCMM-22-2231-s001.docx]

**Supplementary FigS1 Cis-acting elements sequences used in this study**

**hCMV intron A sequence (814bp)**

GTAAGTACCGCCTATAGACTCTATAGGCACACCCCTTTGGCTCTTATGCATGCTATACTGTTTTTGGCTTGGGGCCTATACACCCCCGCTCCTTATGCTATAGGTGATGGTATAGCTTAGCCTATAGGTGTGGGTTATTGACCATTATTGACCACTCCCCTATTGGTGACGATACTTTCCATTACTAATCCATAACATGGCTCTTTGCCACAACTATCTCTATTGGCTATATGCCAATACTCTGTCCTTCAGAGACTGACACGGACTCTGTATTTTTACAGGATGGGGTCCCATTTATTATTTACAAATTCACATATACAACAACGCCGTCCCCCGTGCCCGCAGTTTTTATTAAACATAGCGTGGGATCTCCACGCGAATCTCGGGTACGTGTTCCGGACATGGGCTCTTCTCCGGTAGCGGCGGAGCTTCCACATCCGAGCCCTGGTCCCATGCCTCCAGCGGCTCATGGTCGCTCGGCAGCTCCTTGCTCCTAACAGTGGAGGCCAGACTTAGGCACAGCACAATGCCCACCACCACCAGTGTGCCGCACAAGGCCGTGGCGGTAGGGTATGTGTCTGAAAATGAGCTCGGAGATTGGGCTCGCACCGTGACGCAGATGGAAGACTTAAGGCAGCGGCAGAAGAAGATGCAGGCAGCTGAGTTGTTGTATTCTGATAAGAGTCAGAGGTAACTCCCGTTGCGGTGCTGTTAACGGTGGAGGGCAGTGTAGTCTGAGCAGTACTCGTTGCTGCCGCGCGCGCCACCAGACATAATAGCTGACAGACTAACAGACTGTTCCTTTCCATGGGTC

**hCMV exon+intron A sequence (935bp)** TCAGATCGCCTGGAGACGCCATCCACGCTGTTTTGACCTCCATAGAAGACACCGGGACCGATCCAGCCTCCGCGGCCGGGAACGGTGCATTGGAACGCGGATTCCCCGTGCCAAGAGTGACGTAAGTACCGCCTATAGACTCTATAGGCACACCCCTTTGGCTCTTATGCATGCTATACTGTTTTTGGCTTGGGGCCTATACACCCCCGCTCCTTATGCTATAGGTGATGGTATAGCTTAGCCTATAGGTGTGGGTTATTGACCATTATTGACCACTCCCCTATTGGTGACGATACTTTCCATTACTAATCCATAACATGGCTCTTTGCCACAACTATCTCTATTGGCTATATGCCAATACTCTGTCCTTCAGAGACTGACACGGACTCTGTATTTTTACAGGATGGGGTCCCATTTATTATTTACAAATTCACATATACAACAACGCCGTCCCCCGTGCCCGCAGTTTTTATTAAACATAGCGTGGGATCTCCACGCGAATCTCGGGTACGTGTTCCGGACATGGGCTCTTCTCCGGTAGCGGCGGAGCTTCCACATCCGAGCCCTGGTCCCATGCCTCCAGCGGCTCATGGTCGCTCGGCAGCTCCTTGCTCCTAACAGTGGAGGCCAGACTTAGGCACAGCACAATGCCCACCACCACCAGTGTGCCGCACAAGGCCGTGGCGGTAGGGTATGTGTCTGAAAATGAGCTCGGAGATTGGGCTCGCACCGTGACGCAGATGGAAGACTTAAGGCAGCGGCAGAAGAAGATGCAGGCAGCTGAGTTGTTGTATTCTGATAAGAGTCAGAGGTAACTCCCGTTGCGGTGCTGTTAACGGTGGAGGGCAGTGTAGTCTGAGCAGTACTCGTTGCTGCCGCGCGCGCCACCAGACATAATAGCTGACAGACTAACAGACTGTTCCTTTCCATGGGTC

**TPL intron sequence (520bp)**

TCCTCACTCTCTTCCGCATCGCTGTCTGCGAGGGCCAGCTGTTGGGCTCGCGGTTGAGGACAAACTCTTCGCGGTCTTTCCAGTACTCTTGGATCGGAAACCCGTCGGCCTCCGAACGGTACTCCGCCACCGAGGGACCTGAGCGAGTCCGCATCGACCGGATCGGAAAACCTCTCGAGAAAGGCGTCTAACCAGTCACAGTCGCAAGGTAGGCTGAGCACCGTGGCGGGCGGCAGCGGGTGGCGGTCGGGGTTGTTTCTGGCGGAGGTGCTGCTGATGATGTAATTAAAGTAGGCGGTCTTGAGACGGCGGATGGTCGAGGTGAGGTGTGGCAGGCTTGAGATCCAGCTGTTGGGGTGAGTACTCCCTCTCAAAAGCGGGCATTACTTCTGCGCTAAGATTGTCAGTTTCCAAAAACGAGGAGGATTTGATATTCACCTGGCCCGATCTGGCCATACACTTGAGTGACAATGACATCCACTTTGCCTTTCTCTCCACAGGTGTCCACTCCCAGGTCCAA

**SV40 intron sequence (99bp)**

GTAAGTTTAGTCTTTTTGTCTTTTATTTCAGGTCCCGGATCCGGTGGTGGTGCAAATCAAAGAACT GCTCCTCAGTGGATGTTGCCTTTACTTCTAGGC

**CFEF1 gene intron 1 sequence (943bp)**

GTGAGTGGCGGGTGTGGCCTCCGCGGGCCCGGGCTCCCTCCTTTGAGCGGGGTCGGACCGCCGTGCGGGTGTCGTCGGCCGGGCTTCTCTGCGAGCGTTCCCGCCCTGGATGGCGGGCTGTGCGGGAGGGCGAGGGGGGGAGGCCTGGCGGCGGCCCCGGAGCCTCGCCTCGTGTCGGGCGTGAGGCCTAGCGTGGCTTCCGCCCCGCCGCGTGCCACCGCGGCCGCGCTTTGCTGTCTGCCCGGCTGCCCTCGATTGCCTGCCCGCGGCCCGGGCCAACAAAGGGAGGGCGTGGAGCTGGCTGGTAGGGAGCCCCGTAGTCCGCATGTCGGGCAGGGAGAGCGGCAGCAGTCGGGGGGGGGACCGGGCCCGCCCGTCCCGCAGCACATGTCCGACGCCGCCTGGACGGGTAGCGGCCTGTGTCCTGATAAGGCGGCCGGGCGGTGGGTTTTAGATGCCGGGTTCAGGTGGCCCCGGGTCCCGGCCCGGTCTGGCCAGTACCCCGTAGTGGCTTAGCTCCGAGGAGGGCGAGCCCGCCCGCCCGGCACCAGTTGCGTGCGCGGAAAGATGGCCGCTCCCGGGCCCTGTAGCAAGGAGCTCAAAATGGAGGACGCGGCAGCCCGGCGGAGCGGGGCGGGTGAGTCACCCACACAAAGGAAGAGGGCCTTGCCCCTCGCCGGCCGCTGCTTCCTGTGACCCCGTGGTGTACCGGCCGCACTTCAGTCACCCCGGGCGCTCTTTCGGAGCACCGCTGGCCTCCGCTGGGGGAGGGGATCTGTCTAATGGCGTTGGAGTTTGCTCACATTTGGTGGGTGGAGACTGTAGCCAGGCCAGCCTGGCCATGGAAGTAATTCTTGGAATTTGCCCATTTTGAGTTTGGAGCGAAGCTGATTGACAAAGCTGCTTAGCCGTTCAAAGGTATTCTTCGAACTTTTTTTTTAAG

**IVS intron** **sequence (296bp)**

GAATTAATTCGCTGTCTGCGAGGGCCGGCTGTTGGGGTGAGTACTCCCTCTCAAAAGCGGGCATGACTTCTGCGCTAAGATTGTCAGTTTCCAAAAACGAGGAGGATTTGATATTCACCTGGCCCGCGGTGATGCCTTTGAGGGTGGCCGCGTCCATCTGGTCAGAAAAGACAATCTTTTTGTTGTCAAGCTTGAGGTGTGGCAGGCTTGAGATCTGGCCATACACTTGAGTGACAATGACATCCACTTTGCCTTTCTCTCCACAGGTGTCCACTCCCAGGTCCAACTGCAGGTCG
